# Conserved Noncoding Sequences Regulate *lhx5* Expression in the Zebrafish Forebrain

**DOI:** 10.1371/journal.pone.0132525

**Published:** 2015-07-06

**Authors:** Liu Sun, Fengjiao Chen, Gang Peng

**Affiliations:** Institutes of Brain Science, State Key Laboratory of Medical Neurobiology and Collaborative Innovation Center for Brain Science, Fudan University, Shanghai, China; CNRS, UMR 9197, FRANCE

## Abstract

The LIM homeobox family protein Lhx5 plays important roles in forebrain development in the vertebrates. The *lhx5* gene exhibits complex temporal and spatial expression patterns during early development but its transcriptional regulation mechanisms are not well understood. Here, we have used transgenesis in zebrafish in order to define regulatory elements that drive *lhx5* expression in the forebrain. Through comparative genomic analysis we identified 10 non-coding sequences conserved in five teleost species. We next examined the enhancer activities of these conserved non-coding sequences with Tol2 transposon mediated transgenesis. We found a proximately located enhancer gave rise to robust reporter EGFP expression in the forebrain regions. In addition, we identified an enhancer located at approximately 50 kb upstream of *lhx5* coding region that is responsible for reporter gene expression in the hypothalamus. We also identify an enhancer located approximately 40 kb upstream of the *lhx5* coding region that is required for expression in the prethalamus (ventral thalamus). Together our results suggest discrete enhancer elements control *lhx5* expression in different regions of the forebrain.

## Introduction

The LIM homeobox family gene *lhx5* encodes a protein with two cysteine-rich LIM domains and a homeobox domain [1]. Lhx5 protein is a regulator of central nervous system development [2]. *LHX5* gene sequence variations and copy number variants have been investigated for their association with bipolar disorder and schizophrenia [3, 4]. A fundamental role of *Lhx5* in hippocampal morphogenesis and neuronal differentiation has been demonstrated in mice [5]. In addition, conditional *Lhx5*-deficiency causes learning impairment and motor dysfunction in adult mice [6]. *Lhx5* regulates development processes in multiple brain regions [7–13]. Previous studies also suggest *lhx5* regulate differential adhesion of early ectodermal cells in *Xenopus* [14], promote forebrain development and inhibit Wnt signaling in zebrafish [15], and regulate neural retina development in chicken [16].

The *lhx5* gene has complex temporal and spatial expression patterns during embryonic development. *LHX5* transcripts are detected in human fetal brain and in various regions of adult central nervous system including the spinal cord, thalamus, and cerebellum [17]. In mouse, *Lhx5* expression is detected in the most anterior portion of the neural tube at the headfold stage. After neural tube closure, *Lhx5* is expressed within diencephalic primordium. By mid-gestation, *Lhx5* is expressed in the diencephalon and ventral telencephalon. *Lhx5* is also expressed in the midbrain, hindbrain, and spinal cord after E10.5 of gestation [18]. Compared to mouse, *Xenopus lhx5* is expressed in entire ectoderm in early gastrula embryo. During neurulation, expression of the *lhx5* gene is rapidly restricted to an anterior region in the developing neural plate/keel. In 2-day old Xenopus embryo, this region is more sharply defined, forming a strongly *lhx5*-expressing domain in the diencephalon anterior to the midbrain-forebrain boundary [19, 20]. Zebrafish *lhx5* expression patterns resemble those of Xenopus and by the segmentation stage, zebrafish *lhx5* transcripts are detected in the telencephalon, diencephalon, and discrete regions in the hypothalamus and hindbrain [19].

Despite these broadly conserved expression patterns of *lhx5* genes, the mechanisms underlying *lhx5* transcriptional regulation are not well understood. We showed previously a modified bacteria artificial chromosome (BAC) transgenic zebrafish *lhx5* line recapitulated endogenous *lhx5* expression patterns [21, 22]. Thus the transcriptional regulatory elements are contained in the genomic sequences carried by the BAC, which has approximately 200kb of zebrafish genomic sequences. In this study, we carry out comparative genomic analyses of *lhx5* genomic sequences from various vertebrate species. We find multiple conserved non-coding sequences (CNSs) among different species. We further examine the transcriptional regulatory activity of these CNSs by transient and stable transgenic methods in the zebrafish model.

## Results

### Conserved syntenies in the *lhx5* loci

**Fig 1:**
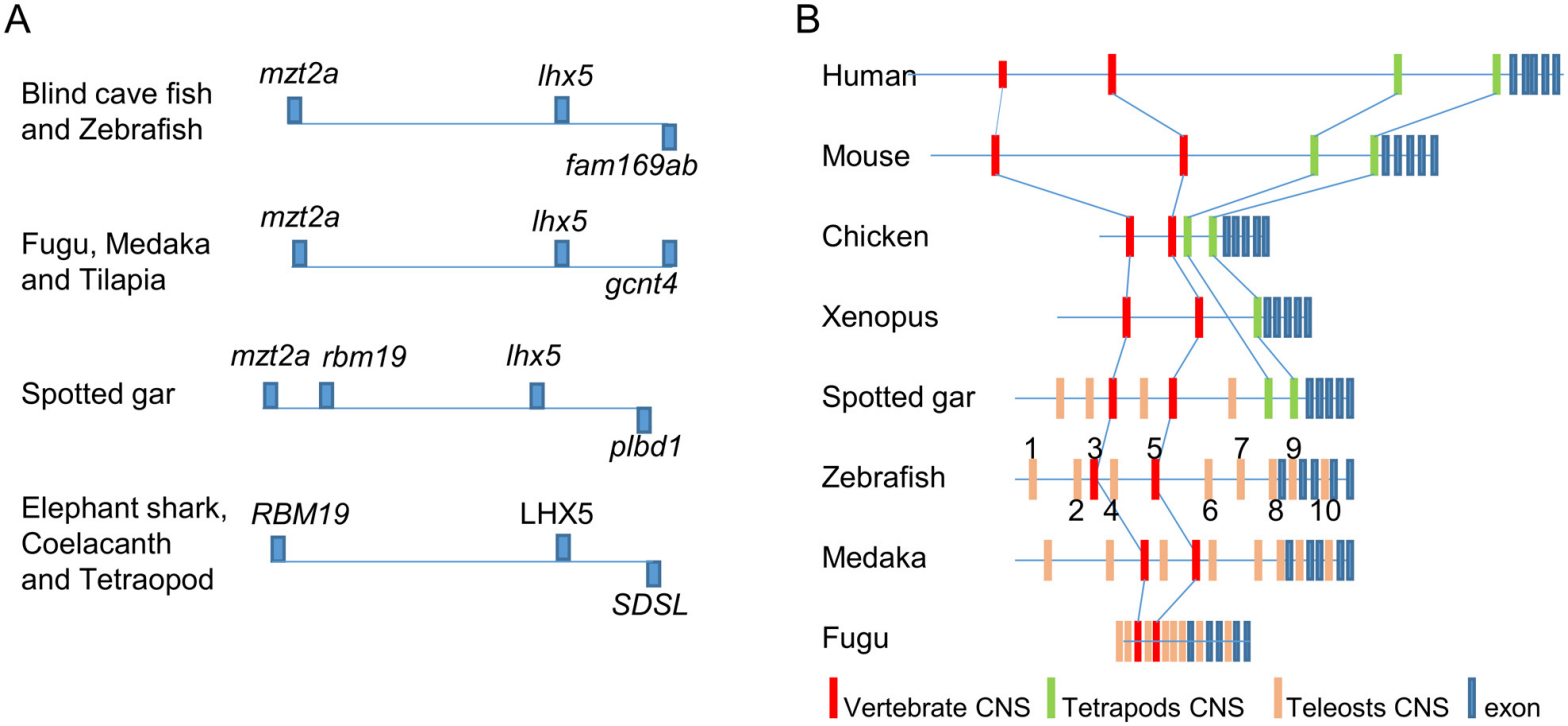
Schematic overview of the *lhx5* locus. (**A**) Genomic regions surrounding *lhx5* genes from vertebrate species. The teleost *lhx5* gene is flanked by adjacent upstream gene *mzt2b* (Fugu, Medaka, Tilapia, cavefish and zebrafish). The cartilaginous elephant shark, the lobe-finned coelacanth, and the tetrapod species show the same *rbm19*-*lhx5*-*sdsl* gene co-localization. The spotted gar *mzt2b*-*rbm19*-*lhx5* gene co-localization is partially similar to both the tetrapod and the teleost fishes. (**B**) Conserved non-coding sequences (CNS) in the upstream genomic sequence of *lhx5* locus in eight representative vertebrate species. Eight of the ten CNSs are teleost-specific, and two CNSs (CNS3 and CNS5) are found in all eight species.

Conserved non-coding sequences (CNSs) are evolutionary conserved intergenic or intronic sequence elements derived from a common ancestor. We carried out comparative sequence analysis of the *lhx5* loci to look for CNSs with putative roles in *lhx5* gene transcriptional regulation. Twelve vertebrate species representing major groups of the jawed vertebrate lineage were included in our analyses, including genomic sequences from human, mouse, chicken, frog, coelacanth, elephant shark, spotted gar, and five teleost fishes (Fig 1). The shared synteny analyses indicated *lhx5* genomic regions in zebrafish and other teleost species showed conserved syntenies. In all teleosts, the *mzt2a* gene is located upstream to the *lhx5* gene. The *LHX5* loci in tetrapods didn’t show conserved synteny with the corresponding regions in the teleost fishes. Nevertheless, conserved syntenies were observed between the tetrapods and other fish species (Fig 1A). Thus the cartilaginous elephant shark and the lobe-finned coelacanth showed the same *rbm19*-*lhx5*-*sdsl* gene co-localization as in the tetrapods. Interestingly, the spotted gar *mzt2b*-*rbm19*-*lhx5* gene co-localization was partially similar to both the tetrapod and the teleost fishes. These conserved synteny results suggested the genomic sequences in the *lhx5* regions were conserved through evolution but speciation in teleost lineage has diversified the gene co-localizations.

### Conserved non-coding sequences in the *lhx5* genomic regions

Based on the shared syntenies between spotted gar, tetrapods, and the teleosts, we used the spotted gar as intermediate base species to compare vertebrate *lhx5* gene upstream genomic sequences [23]. We performed multiple sequence alignments with MultiPipmaker and mVISTA programs, using a window size of 100 bp and similarity threshold of 70%. Results from both programs were in good agreements. Comparison between the cartilaginous fish, ray-finned fishes, lobe-finned fish and tetrapod identified two common CNSs in all 12 species (Fig 1B). In addition, two more non-exonic sequence elements that qualified as CNSs were identified in the four tetrapod species, and eight CNSs were found among the five teleost species. Interestingly, the spotted gar *lhx5* genomic region contained the two additional tetrapod specific CNSs and four of the eight teleost specific CNSs, in addition to the two common CNSs conserved in all 12 species (Fig 1B). A VISTA plot showing the sequence alignment of the *lhx5* loci from the five teleost species is shown in the Supplmentary Figures (S1 Fig).

### Region specific enhancer activity of the identified CNSs

In the zebrafish genome, the 10 identified teleost specific CNSs were located to a region spanning approximately 50 kb. Two CNSs resided in the first and the third intron of the *lhx5* gene, respectively. One CNS was located in the *lhx5* promoter region, within 1 kb from the *lhx5* transcriptional start site. Other CNSs were located in the upstream distal intragenic regions away from the transcriptional start site. These 10 CNSs were named based on their positions with respect to the *lhx5* coding regions as CNS1 to CNS10. The lengths of these CNSs were around 200bp, except for CNS4, which spanned approximately 800 bp (S1 Table).

We first carried out transient reporter EGFP assay to examine tissue specific enhancer activities of these CNS elements. Each of the ten CNSs was PCR amplified and cloned in a vector carrying a basal promoter [24] and a modified Gal4 transcriptional activator coding sequence [25]. In order to maintain the endogenous genomic milieu of these CNS elements in the reporter constructs, the average size of the amplified fragments was around 3Kb and contained genomic sequences surrounding the identified CNS elements (S2 Table).

**Fig 2:**
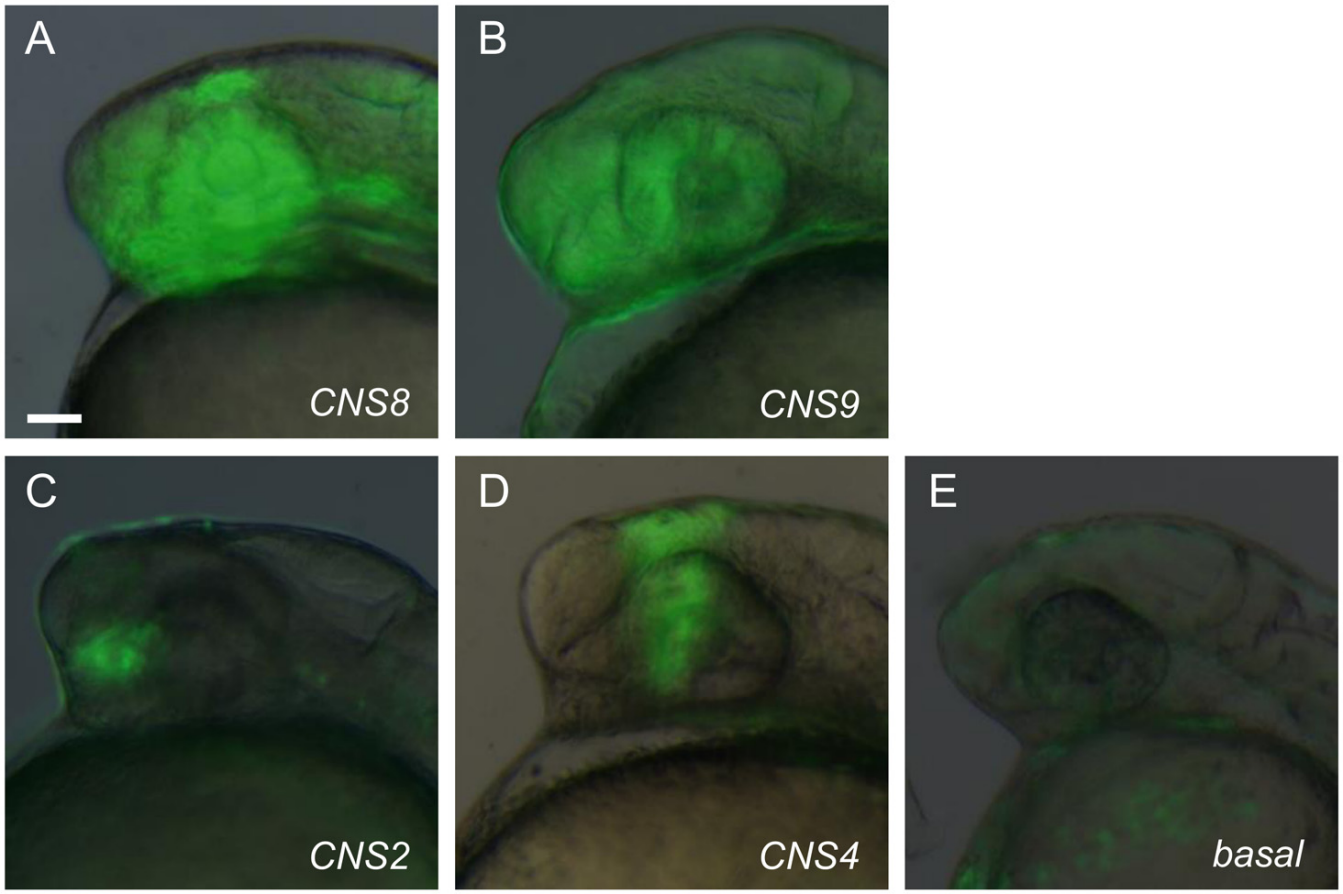
Region specific enhancer activity of the identified CNSs. (**A-B**) CNS8 and CNS9, located in the vicinity of the *lhx5* promoter region give rise to broad reporter EGFP expression in the forebrain regions. (**C**) CNS2 located approximately 50 kb upstream of the *lhx5* coding region gives rise to restricted EGFP signal in the anterior ventral forebrain. (**D**) CNS4 located 40 kb upstream of the *lhx5* coding region, gives rise to restricted EGFP expression in the diencephalic region. (**E**) Vector construct gives rise to basal non-tissue specific EGFP expression in transient expression assay. Lateral view of the forebrain regions of embryos at 24 hpf, anterior to the left. Scale bar: 50μm.

We then injected each construct into one-cell stage embryos obtained from a UAS:EGFP report line fish [25]. Three independent microinjections were performed for each of the 10 CNS constructs, and the fluorescent signals were examined at 24, 48 and 72 hours post fertilization (hpf). These transient reporter EGFP assays indicated CNS2, CNS4, CNS8, and CNS9 had tissue specific enhancer activities. While the CNS8 and CNS9 constructs gave rise to broad reporter EGFP expression in the forebrain regions, the CNS2 and CNS4 constructs produced more restricted EGFP expression in the forebrain (data not shown). In contrast, the construct carrying the basal promoter produced little or weak non-tissue specific fluorescent signals (Fig 2E). It should be noted that the CNS8 construct likely also carried basal promoter sequences from the endogenous *lhx5* gene due to proximity of the CNS8 element and the *lhx5* transcription start site. Thus it was unclear whether the CNS8 element contained region specific enhancer activity by itself.

We next used Tol2-based transgenesis to investigate the four CNSs enhancer activities in stable transgenic animals. At least two independent stable transgenic lines with common expression patterns were established for each of the CNS constructs (S2 Fig). Consistent with the transient reporter assays, at 24hpf the two proximately located CNS8 (likely together with *lhx5* basal promoter) and CNS9 both gave rise to robust reporter EGFP expression in the forebrain regions including the telencephalon and the retina (Fig 2A and 2B). These gene expression patterns were much broader than the endogenous expression of *lhx5*. The CNS2 element, which is located 50 kb upstream of the *lhx5* coding regions, gave rise to restricted EGFP expression in the ventral forebrain; whereas the CNS4, located 40 kb upstream of the lhx5 coding region, gave rise to restricted EGFP signal in the diencephalic region (Fig 2C and 2D).

### CNS2 contains hypothalamic enhancer activity and responses to FGF signaling

**Fig 3:**
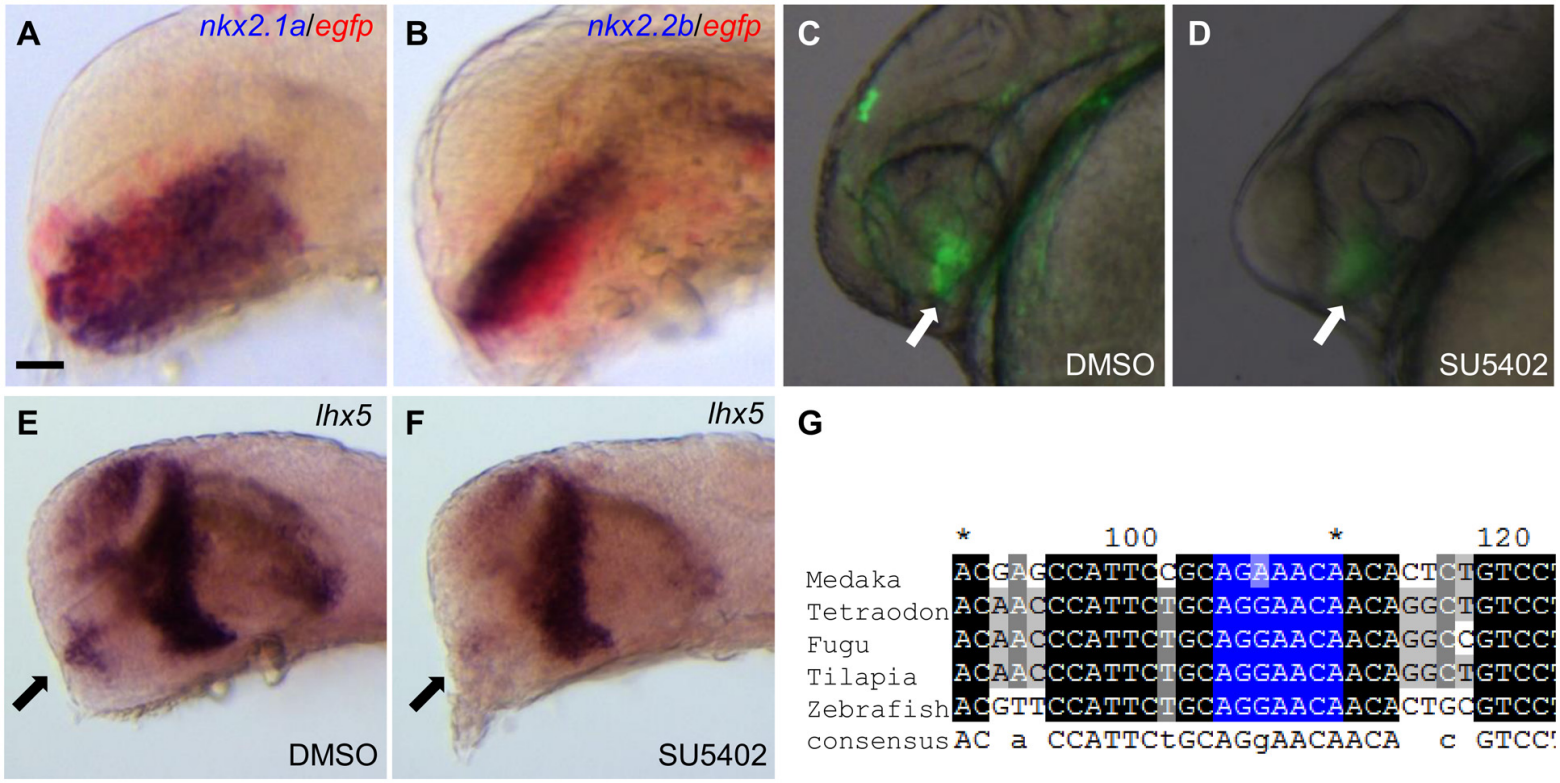
CNS2 contains hypothalamic enhancer activity and responses to FGF signaling. (**A-B**) Double in situ hybridization results indicate CNS2 contains hypothalamic enhancer activity. The hypothalamic marker *nkx2*.*1a* and *nkx2*.*2b* are stained in dark blue, reporter *egfp* stained in red. (**C-D**) SU5402 treatment severely reduces CNS2 activity. Vehicle DMSO treated embryos show restricted hypothalamic EGFP reporter expression (pointed by the arrow in C). Embryos treated with the FGF signaling inhibitor SU5402 during the segmentation stage (10-24hpf) show minimal EGFP signals in the hypothalamic region (arrow in D, n = 48/55). (**E-F**) SU5402 treatment down-regulates endogenous *lhx5* expression in the hypothalamic region. Endogenous *lhx5* shows robust expression in the hypothalamic region (pointed by the arrow in E). SU5402 treatment during the segmentation stage down-regulates *lhx5* expression in the hypothalamic region (arrow in F, n = 25/28). (**G**) Multiple sequence alignments of the CNS2 region in the five teleost species. The identified FGF downstream factor Pea3 binding site is highlighted in blue. Lateral view of the forebrain regions of embryos at 24 hpf (A-F), anterior to the left. Scale bar: 40μm in A-B; 50μm in C-D.

We used molecular markers to further examine the reporter EGFP expression in animals carrying the CNS2 enhancer element. *nkx2*.*1a* and *nkx2*.*2b* are two transcription factors that are specifically expressed in the ventral forebrain, in the hypothalamic regions at 24 hpf. Two-color *in situ* hybridization results showed reporter EGFP and the two *nkx* markers were co-expressed in the hypothalamic regions. The region expressing *nkx2*.*1a* was broader than the EGFP expressing region, whilst the *nkx2*.*2b* expressing region almost overlapped the EGFP expressing region in the hypothalamus (Fig 3A and 3B).

Signaling pathways controlling forebrain patterning and regionalization may regulate transcription factor expression. It was previously reported that FGF signaling pathway was involved in hypothalamus development [26–29]. To examine the likely signaling pathways involved in the regulation of the CNS2 hypothalamic enhancer activity, we used small molecule drug SU5402 to inhibit the FGF signaling. When FGF signaling was inhibited by SU5402 treatment in embryos carrying the CNS2 enhancer element during the segmentation stage (between 10 hpf and 24 hpf), the EGFP expression in the hypothalamic region was severely reduced comparing to the vehicle DMSO treated controls (Fig 3C and 3D). Inhibiting the FGF signaling also abolished endogenous *lhx5* expression in the hypothalamic regions (Fig 3E and 3F). We searched for potential transcription factor binding sites in the CNS2 element using rVISTA 2.0 program and found a conserved *pea3* binding site in the CNS2 from the five teleost species (Fig 3G). Due to possible pleiotropic effects of FGF signaling inhibition on embryonic development, it is not known whether FGF signaling pathway may act directly through the *pea3* binding site within the CNS2 sequence to influence the CNS2 enhancer activity.

### CNS4 relies upon a *zic* binding site to drive pre-thalamic expression

**Fig 4:**
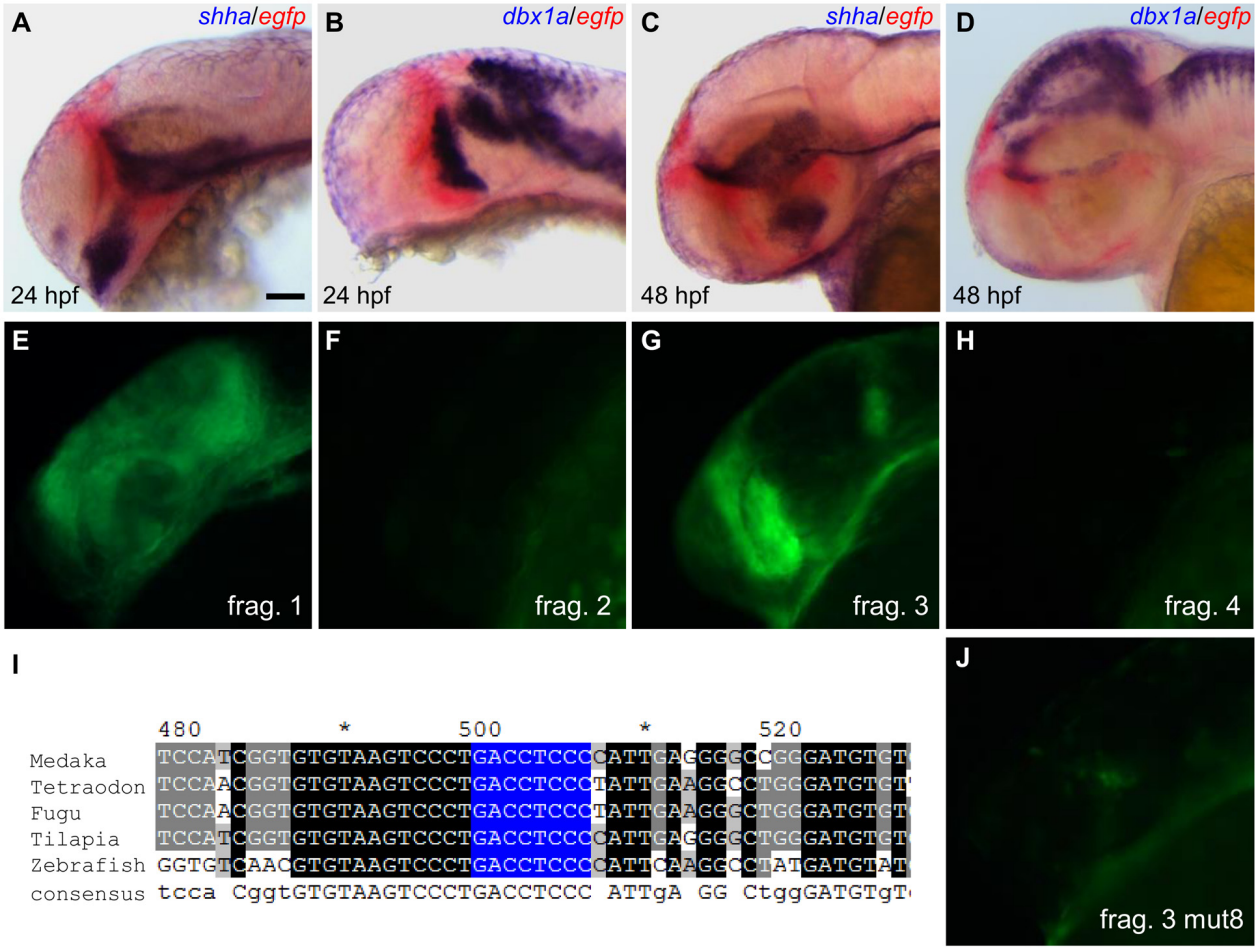
CNS4 contains pre-thalamic enhancer activity and relies on a *zic* binding site. **(A-D)** Double in situ hybridization results indicate CNS4 contains pre-thalamic enhancer activity. ZLI (zona limitans intrathalamica) marker *shha* and thalamic marker *dbx1a* are stained in dark blue, reporter *egfp* stained in red. CNS4 driven EGFP expression abuts anteriorly to the ZLI and thalamus in the 24hpf (A-B) and 48hpf (C-D) stage embryos. (**E-H**) The third fragment of the four subdivisions of the CNS4 sequence contains pre-thalamic enhancer activity. (**I**) Multiple sequence alignments of the CNS4 region in the five teleost species. The identified *zic* binding site is highlighted in blue. (**J**) Mutation of the 8bp *zic* binding site in the prethalamic enhancer abolished its activity. Lateral view of the forebrain regions of embryos at 24 hpf (A-B, E-H, and J) or 48 hpf (C-D), anterior to the left. Scale bar: 50μm.

Two-color in-situ hybridization results showed that reporter EGFP expression in embryos carrying CNS4 element was located anteriorly to the *shha* and *dbx1a* expression in the diencephalic region (Fig 4A to 4D). Thus the CNS4 elements contained pre-thalamic enhancer activity.

The pre-thalamic CNS4 enhancer spanned approximately 800 bp. It was the longest fragment in all identified CNSs in our study. The other CNSs were around 200 bp long. We next made four overlapping 200 bp fragments covering the whole length of CNS4 and we examined the potential enhancer activities of these four smaller elements with Tol2-based transgenesis. The first fragment gave broad low level of reporter EGFP expression. The second and the fourth fragments produced little tissue-specific signals. Interestingly, the third fragment gave rise to the same enhancer activity as the whole CNS4 element (Fig 4E to 4H).

In order to better define the mechanism regulating the third fragment’s enhancer activity in the pre-thalamus, we searched for potential transcription factor binding sites within this fragment by the rVISTA 2.0 program. Through conserved binding site motif search we found a *zic* family member binding site (Fig 4I). Previous study showed the *zic* family member *Zic2a* was involved in the regulation of two prethalamic transcription factor *dlx2a* and *arxa*. [30]. To test whether the *zic* binding site we identified was involved in the regulation of the prethalamic enhancer activity, we made a construct with site-specific mutation at the predicted *zic* binding site (mut8 construct, binding site changed from GACCTCCC to GAAATAAA) and generated stable transgenic animals. The mutated transgenes were mapped to chromosome 13 and chromosome 24 in two independent stable transgenic lines, respectively (S3 Fig). Consistent with the *zic* involvement in the transcriptional regulation of the CNS4, the site-specific mutation construct completely lost the prethalamic enhancer activity (Fig 4J).

## Discussion

In this study, we took advantage of comparative genomics and Tol2 mediated transgenesis in the zebrafish model to examine the transcriptional regulation of the *lhx5* gene. We identified multiple conserved no-coding sequence elements in 12 vertebrate species ranging from the cartilaginous fish to the human. We found three teleost specific CNSs that can drive reporter gene expression in discrete regions in the developing zebrafish forebrain.

### Evolutionally conservation of the regulatory elements of the *lhx5* gene

Vertebrate *lhx5* belongs to the Lhx1/5 subclass of the Lim homeobox gene family, homologous of *C*. *elegans* lin-11 and *D*. *melanogaster* bk87 [2]. All *lhx5* genes shared conserved function during vertebrate CNS patterning and embryonic development [5, 7, 9, 11, 15, 16]. The *lhx5* gene expression patterns also seem largely conserved in the vertebrates [17, 19, 20, 31]. Remarkably, in primitive vertebrate species such as amphioxus, *lhx5* homolog is also expressed in the anterior central nervous systems [32]. The similarity in *lhx5* expression patterns in species ranging from the amphioxus to the mammals suggests *lhx5* expression is based on conserved mechanisms. In the jawed vertebrates lineage, comparative genomic analyses have revealed shared conserved non-coding sequence elements in tetrapods, ray-finned fishes, lobe-finned fishes and cartilaginous fish species [33, 34].

The three region specific enhancer elements identified in our study are conserved in the teleost lineage only. No corresponding CNS elements are found in the tetrapod lineage. Nevertheless, our synteny analysis results and the existence of pan-vertebrate CNSs indicate sequence conservations in the *lhx5* upstream regulatory regions. Therefore, it is possible the corresponding regulatory elements have become too diversified to be recognizable by sequence comparison methods we employed. A previous study showed the regulatory sequences from the human RET locus were able to drive *ret*-specific expression in zebrafish, despite there was no recognizable sequence similarity between the human and zebrafish regulatory elements [35]. In addition, enhancers from the even skipped locus in the scavenger and the vinegar flies did not show sequence similarities but were functionally similar [36].

### *lhx5* expression in the prethalamus

The endogenous *lhx5* gene is expressed in the prethalamus in developing zebrafish brain [37, 38]. Here we showed the CNS4 element of *lhx5* can drive region specific reporter expression in the prethalamus. Interestingly, it has been shown epigenetic marker H3K4me1 is enriched in the CNS4 region during the early development of zebrafish embryos [39]. The enrichment of H3K4me1 modification at the CNS4 region is consistent with its involvement in the transcriptional regulation of the endogenous *lhx5* gene.

Prethalamus is part of the diencephalon. Based on the prosomeric model [40–42], prethalamus corresponds to prosomere 3 (p3). Previous studies have shown within the diencephalon, the patterning events are regulated by the mid-diencephalic organizer (MDO) zona limitans intrathalamica (ZLI) [38, 43]. Hedgehog signal emanating from the ZLI plays essential functions in the prethalamus development [38]. Downstream of the hedgehog signaling pathway, the Zic family transcriptional factors may mediate the effect of hedgehog signal [44, 45]. In the developing zebrafish forebrain between 4 and 8 somite stages, the *zic2a* gene is expressed in the regions fated to become the prethalamus. Thus knockdown of *zic2a* function perturbs the prethalamic development and caused significant reduction of region specific marker expression in the prethalamus [30]. Later in segmentation stages, *zic2a* is no longer expressed in the prethalamic regions and Zic2a may act independently of hedgehog signaling to promote prethalamic development [30]. Interestingly, the *zic3* gene is expressed in the anterior diencephalon during the segmentation and pharyngula stages. A recent study showed Zic3 interaction with distal regulatory elements of stage specific developmental genes including *lhx5* in the zebrafish model [46]. The identified Zic3 binding site lies more upstream of the region we studied, and the sequence of the identified Zic3 binding site is different from the potential *zic* binding site identified in our site-specific mutation analysis. The *zic* binding site identified in our study conforms to the Zic3 consensus binding site determined in a previous report [47].

In conclusion, our study identified multiple conserved non-coding sequence elements in the *lhx5* locus. Three of the teleost specific CNSs can drive reporter gene expression in discrete regions in the developing zebrafish forebrain. Together our results suggest that discrete enhancer elements control *lhx5* expression in different regions of the forebrain.

## Materials and Methods

### Sequence analysis

Sequences for 12 gnathostome *lhx5* loci were downloaded from NCBI RefSeq genome sequences: *Danio rerio* (zebrafish): chromosome 21:14116198..14290982; *Takifugu rubripes* (fugu): chromosome 6:5719292..5770520; *Oryzias latipes* (medaka): chromosome 12:2078644..2245397; *Oreochromis niloticus* (Nile tilapia): NC_022205:11084704..11177152; *Lepisosteus oculatus* (spotted gar): NC_023198:14084503..14284612; *Astyanax mexicanus* (Mexican tetra, blind cave fish): NW_006749407:14473..208111; *Latimeria chalumnae* (coelacanth): NW_005819674:109551..725575; *Gallus gallus* (chicken) chromosome 15:12452471..12575304; *Homo sapiens* (human) chromosome 12:113462889..113966371; *Mus musculus* (house mouse) chromosome 5:120116513..120441457; *Xenopus tropicalis* (western clawed frog) NW_004668232:61749600..61904154; *Callorhinchus milii* (elephant shark) NW_006890147:2032526..2289716. Alignments of long genomic sequences were performed with the mVISTA LAGAN program [48, 49]. For alignments of the conserved region upstream of *lhx5* at the nucleotide level the ClustalX program [50] was used. For the prediction of transcription factor binding sites the rVista 2.0 server [51] with TRANSFAC database was employed (search parameters: TRANSFAC Professional; biological species: vertebrates; matrix similarity: optimized for function) [52].

### Fish maintenance

Zebrafish were maintained in a recirculating water system according to standard protocol [53]. Zebrafish embryos were obtained from mating of adult fish and raised at 28.5°C as described [53]. Embryos were staged by hours post fertilization (hpf) [54]. The Fudan University Institutional Animal Care and Use Committee approved all work with zebrafish animals (project number: 0227–092).

### Vector construction

The backbone plasmid pminiTol2-super-Gal4FF contains minimal Tol2 transposable elements [55] flanking a super core basal promoter [24] and a modified version of Gal4 transcriptional activator [25]. The backbone plasmid was linearized by SmaI restriction digestion and then dephosphorylated by calf intestine phosphatase treatment. DNA fragments containing the conserved non-coding sequences were amplified by PCR with primers listed in S2 Table. The sizes and coordinates of the amplified fragments are listed in S2 Table. The BAC clone DKEY-106C24 (GenBank ACCESSSION: BX569800) was used as the PCR template. PCR products were treated with T4 polynucleotide kinase and then ligated into the linearized backbone vector. Constructs with forward insertion of CNSs were selected by restriction enzyme digestion and sequencing. All constructs were verified by sequencing.

To divide the prethalamic specific CNS4 element into four overlapping fragments, PCR primers listed in S2 Table were used.

To mutate the *zic* binding site, we replace the original binding site sequence GACCTCCC with GAAATAAA via chemical synthesis (Sangon, Shanghai) of the ~200 bp F3 fragment.

### Generation of Tg(*lhx5*:Gal4FF, UAS:EGFP) transgenic line

Generation of transgenic zebrafish was performed essentially as described [56]. Briefly, the constructs carrying *lhx5* CNS were prepared over standard columns then further purified by dialysis. Tol2 transposase mRNA was in vitro transcribed from linearized pCS2-TP plasmid (kindly provided by Prof. Koichi Kawakami) with mMESSAGE mMACHINE SP6 Transcription Kit and then column-purified. For injection, equal volumes of 20 ng/μl of plasmid DNA and 200 ng/μl of transposase mRNA were mixed and 1~2 nl of the mixture was injected into one-cell stage embryos.

To identify transgenic founder fish, 50–100 of embryos injected with a given plasmid were raised to maturity and outcrossed with Tg(UAS:EGFP) reporter line animal [25]. Outcrossed off-springs were analyzed for EGFP expression using a stereo fluorescence microscope (Leica M205FA). Two independent stable transgenic lines with common expression patterns were established for each CNS construct.

### Fluorescence microscopy

Zebrafish embryos were treated with 0.003% 1-phenyl-2-thiourea (PTU) to inhibit pigmentation. Embryos were anesthetized with tricaine, mounted in 3% methyl cellulose, and imaged under a fluorescence microscope (Leica M205FA).

### Whole-mount In situ hybridization

Whole-mount in situ hybridization was performed as described [57]. Digoxigenin-labeled antisense RNA probes were hybridized and then detected with alkaline phosphatase-conjugated digoxigenin antibody Fab fragment (1:7500, Roche) and alkaline phosphatase substrate NBT/BCIP (1:80, Roche). Two color whole-mount in situ hybridization was performed as described [58].

To make probe clones, RT-PCRs were performed with primers listed in S3 Table. The EGFP fragment of pEGFP-N3 (Clontech) plasmid was isolated using the HindIII and NotI sites and subcloned via the same sites into pBlueScript II KS (+) (Stratagene). All clones were verified by sequencing. Whole mount in situ images were acquired on a dissection microscope with a CCD camera (Leica M205FA).

### Drug treatment

To block Fgf signaling, embryos were treated with 20 μM SU5402 (Calbiochem) in embryos medium from 10 hpf to 24 hpf at 28.5°C. Control embryos were treated in embryo medium with DMSO (SU5402 vehicle). Embryos were placed in 12-well plates (30 embryos per well) with 0.5 ml of medium. After drug treatment, embryos were fixed and processed for in situ hybridization.

### Insertion mapping by hiTAIL-PCR

To determine insertion sites of the transgenic enhancer constructs, high efficiency thermal asymmetric interlaced PCR (hiTAIL-PCR) was performed as described [59], with primers listed in S4 Table. In brief, first round PCR was carried out with LAD1-LAD4 primer mixture and mapLa or mapR1 primer. Second round PCR was carried out with AC1 primer and Tag-mapLb or Tag-mapR2 primer. Third round PCR was carried out with AC1 primer and mapLc or mapR3 primer. Final PCR products were sequenced and BLAST search were performed against Ensembl zebrafish GRCz10 genomic sequence build.

## Supporting Information

S1 Fig Sequence alignment of the *lhx5* loci from teleost fish. Vista plot of the aligned sequences. Zebrafish sequence is used as the base. The identified CNSs are labeled on the Vista plot.
(TIFF)

S2 Fig Stable transgenic lines carrying CNS elements. (**A**) Figure panels illustrate established transgenic lines carrying corresponding CNS elements. Line id is indicated on the upper left corner on the figure panel. Lateral view of the forebrain regions of embryos, anterior to the left. (**B**) Text description of the established transgenic lines. The line id used in experiments described in the Result section is indicated.
(TIFF)

S3 Fig Characterization of insertion sites of the *zic* binding site mutation construct in the zebrafish genome. The mutated transgene is inserted at chr13:10057513 and chr24: 1892994 in two independent stable transgenic lines, respectively. The coordinates are based on the Ensembl zebreafish GRCz10 genomic sequence build.
(TIFF)

S1 Table Sequence coordinates of the identified CNS elements. The sequence coordinates on the Ensembl zebreafish GRCz10 genomic sequence build and the BAC DKEY-106C24 sequence are listed. The sequence identities of CNS elements between the GRCz10 build and DKEY-106C24 are also listed.
(XLSX)

S2 Table Primer sequences used in this study. The sequence coordinates and sizes of the cloned fragments are listed on sheet named CNS. The primer used in sub-dividing CNS4 are listed on sheet named CNS4.
(XLSX)

S3 Table Primer sequences used in constructing plasmids for in situ probes. (XLSX)

S4 Table Primer sequences used in the hiTAIL-PCR. (XLSX)
