# Supplementary figures and images for: Conserved Noncoding Sequences Regulate lhx5 Expression in the Zebrafish Forebrain
Source: PLoS One. 2015 Jul 6;10(7):e0132525. doi: 10.1371/journal.pone.0132525 (PMC4492605; doi:10.1371/journal.pone.0132525)

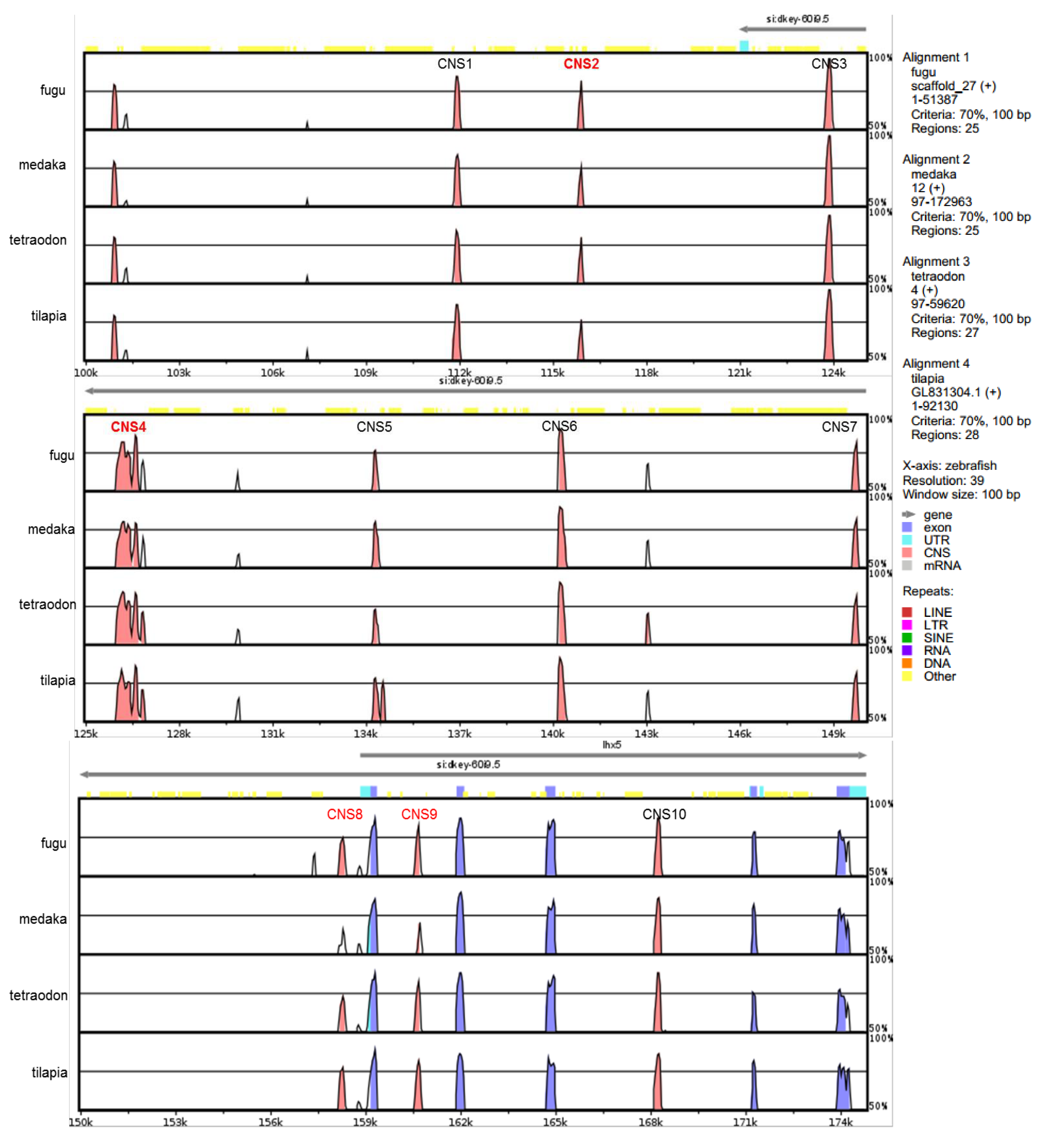

Supplement: S1 Fig — Vista plot of the aligned sequences. Zebrafish sequence is used as the base. The identified CNSs are labeled on the Vista plot. (TIFF) [file pone.0132525.s001.tiff]

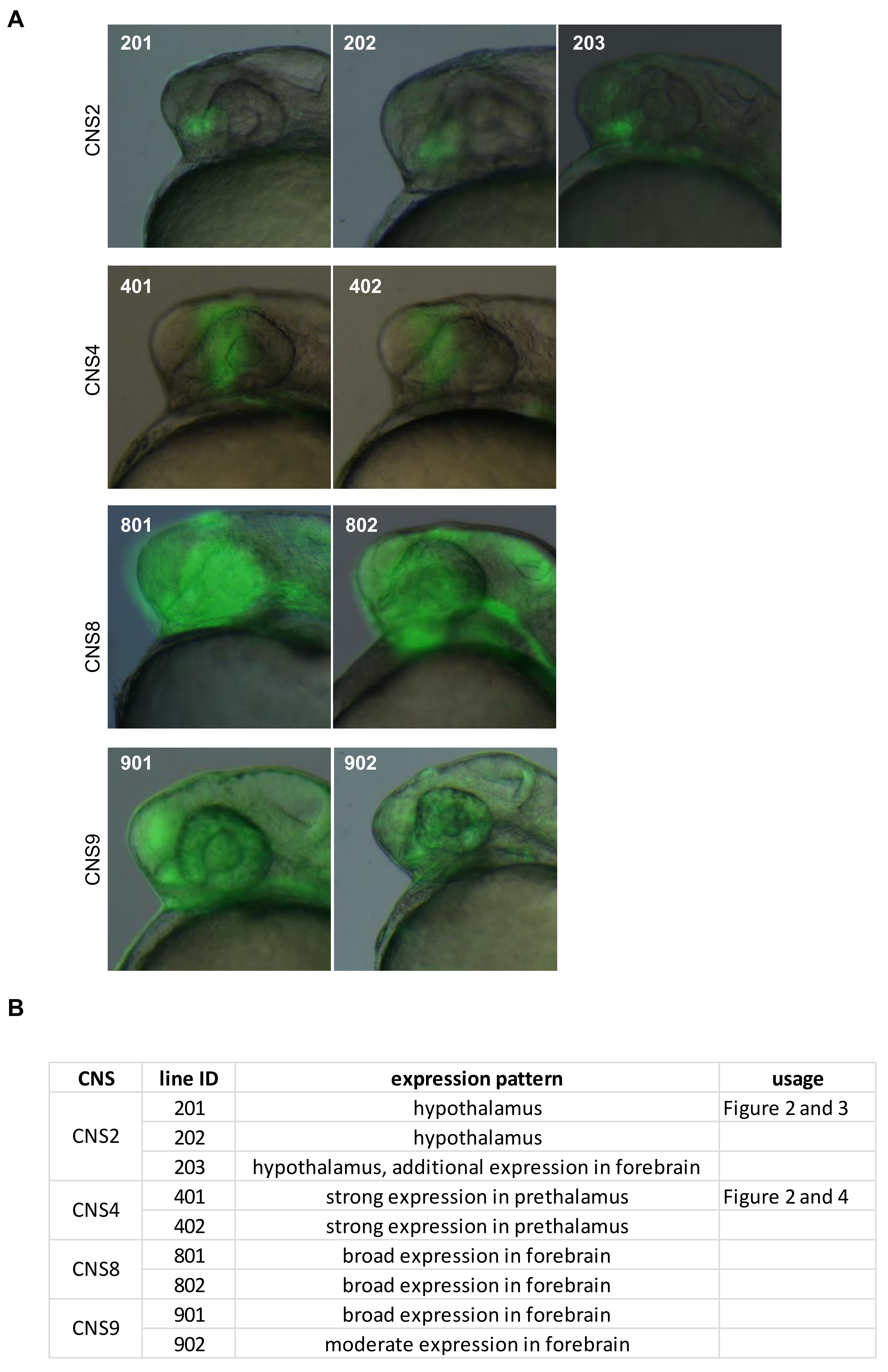

Supplement: S2 Fig — (A) Figure panels illustrate established transgenic lines carrying corresponding CNS elements. Line id is indicated on the upper left corner on the figure panel. Lateral view of the forebrain regions of embryos, anterior to the left. (B) Text description of the established transgenic lines. The line id used in experiments described in the Result section is indicated. (TIFF) [file pone.0132525.s002.tiff]

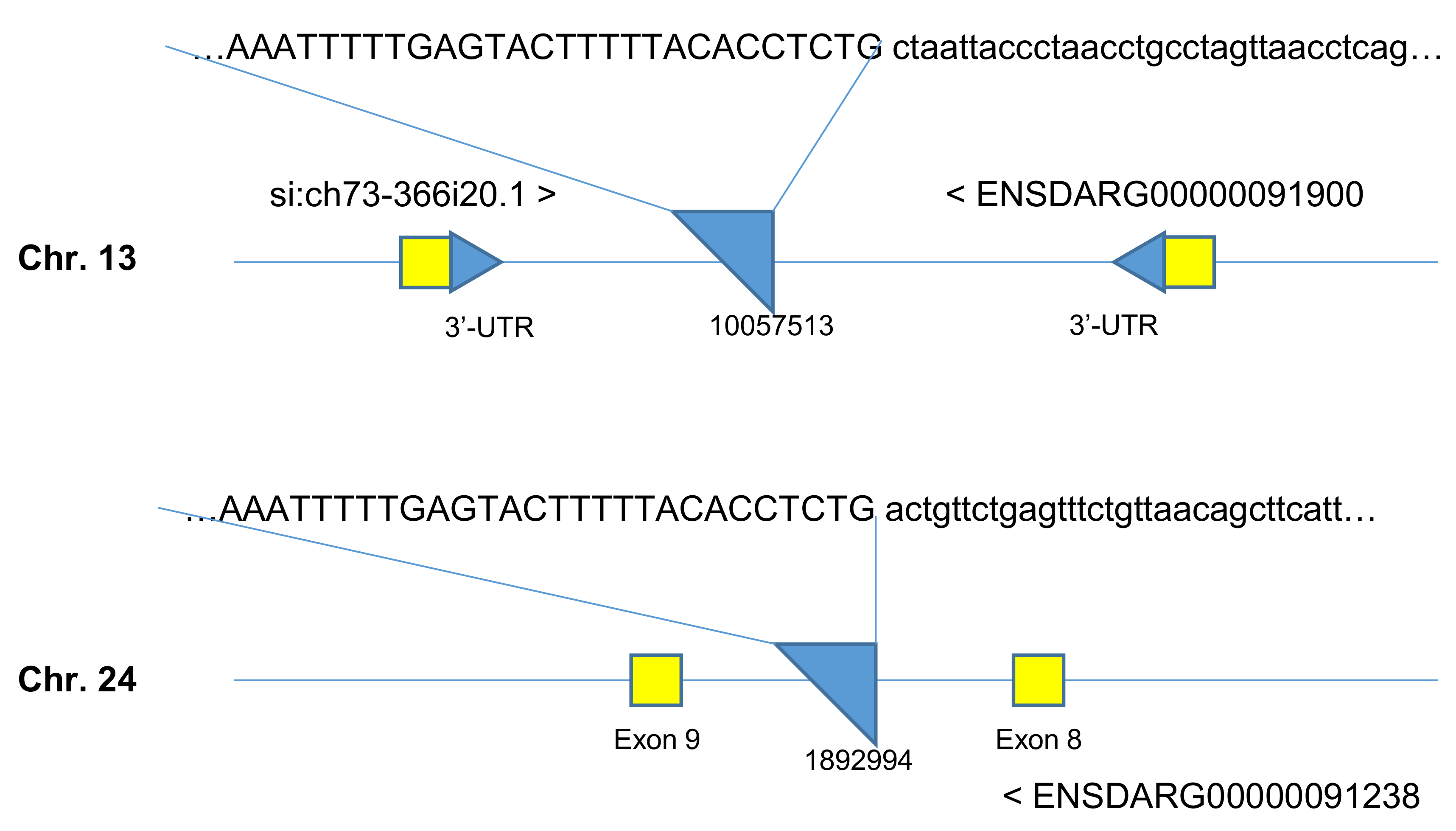

Supplement: S3 Fig — The mutated transgene is inserted at chr13:10057513 and chr24: 1892994 in two independent stable transgenic lines, respectively. The coordinates are based on the Ensembl zebreafish GRCz10 genomic sequence build. (TIFF) [file pone.0132525.s003.TIFF]
